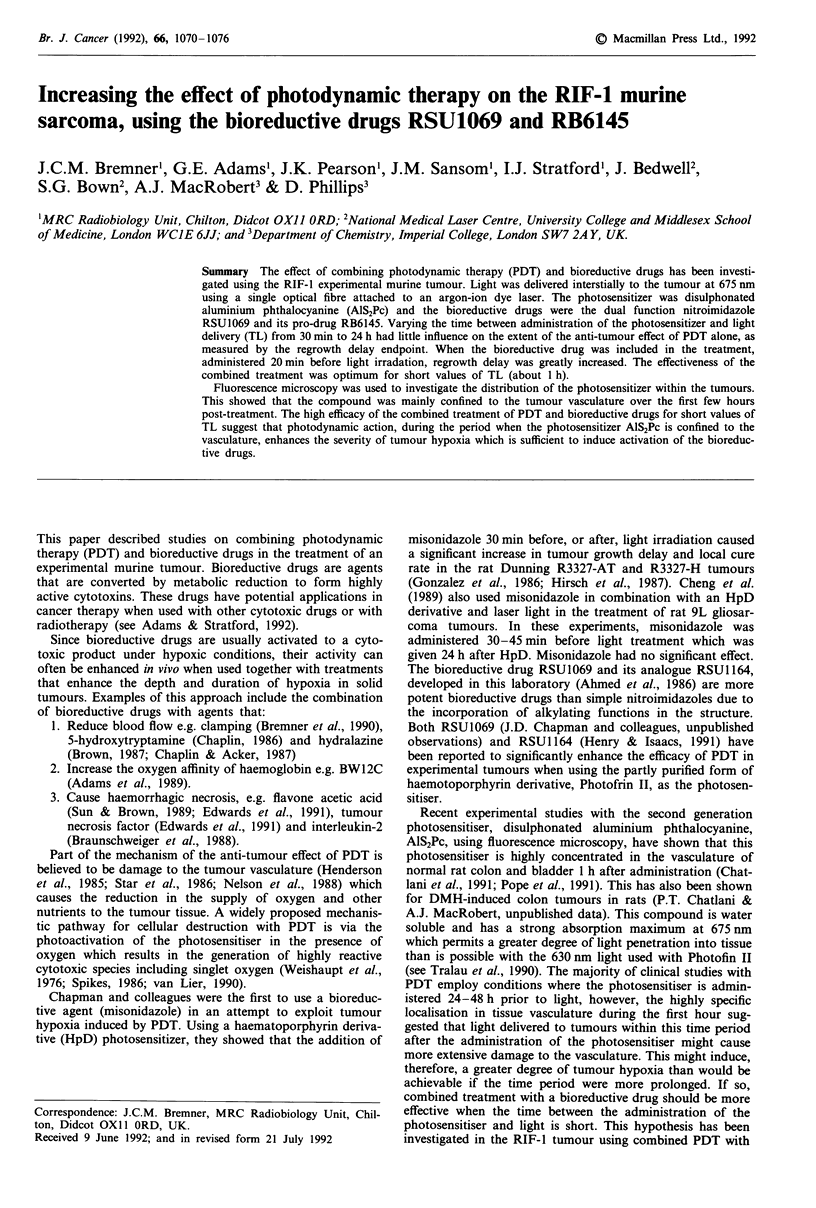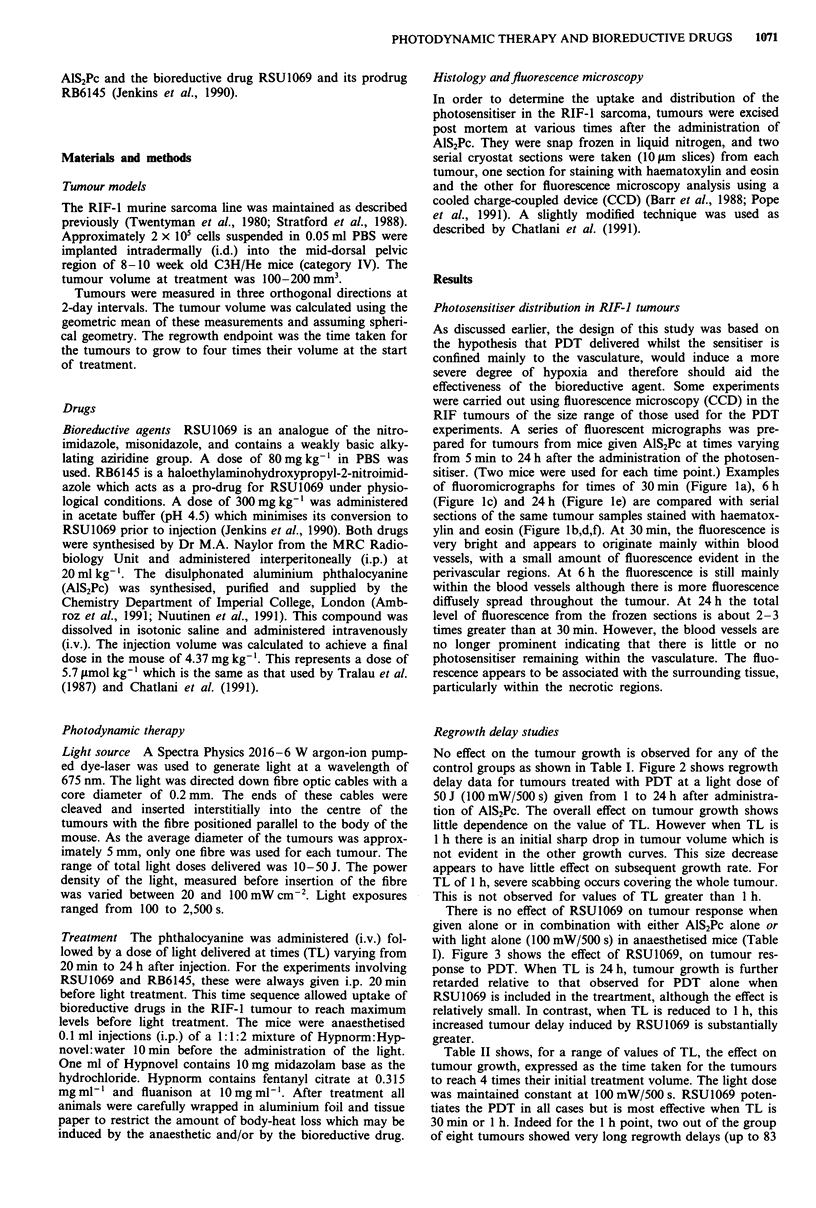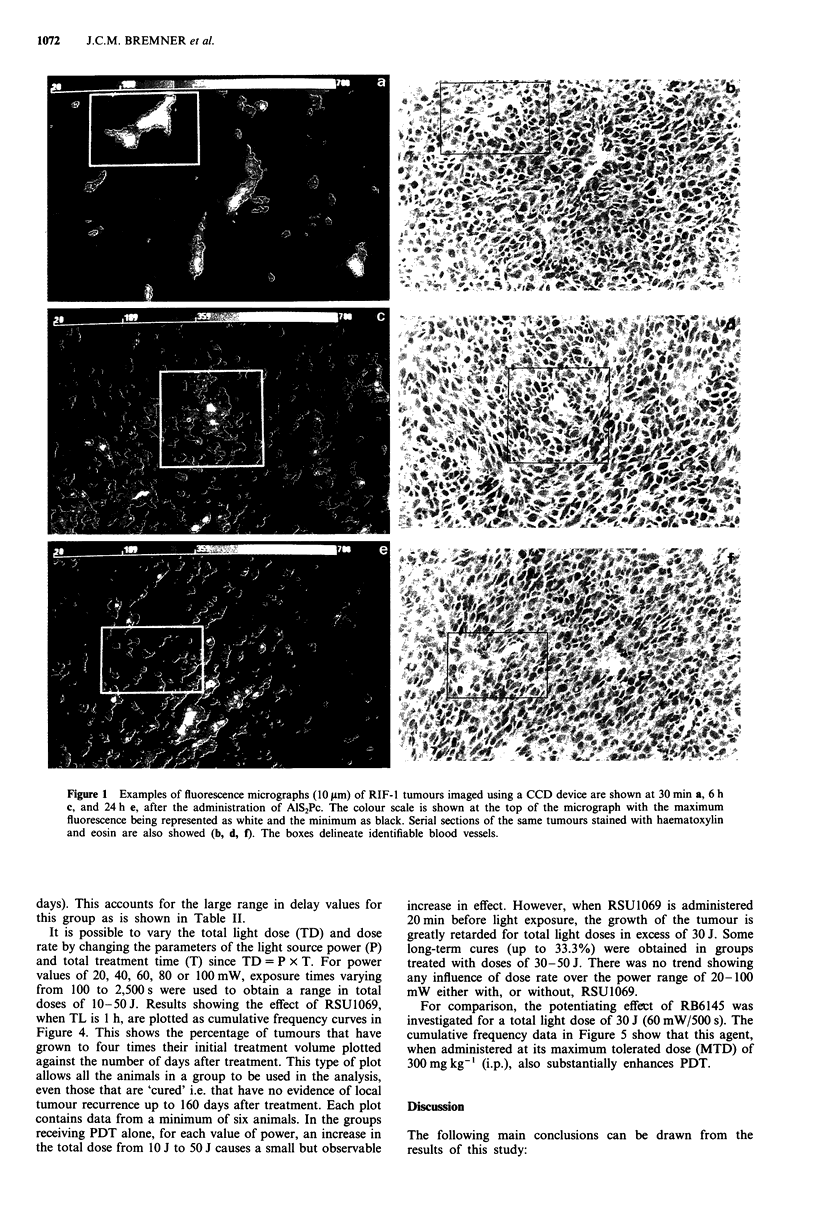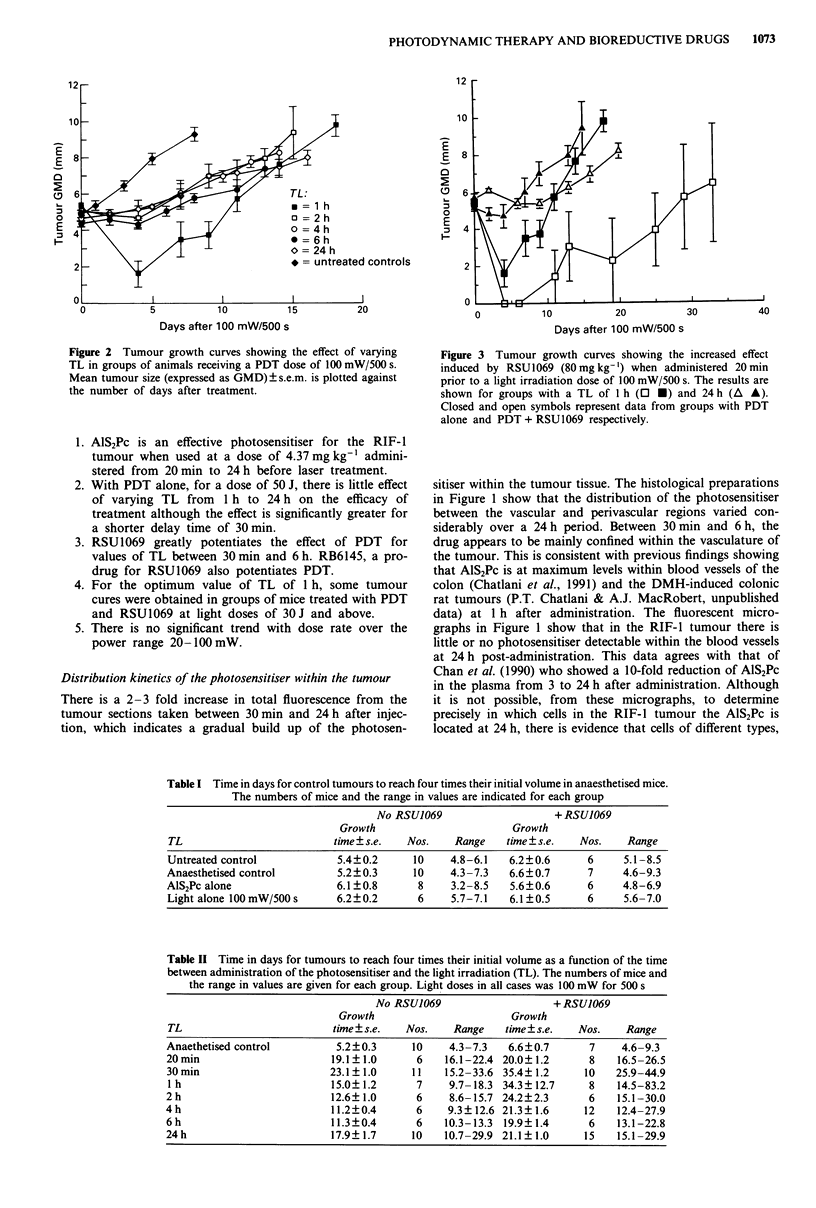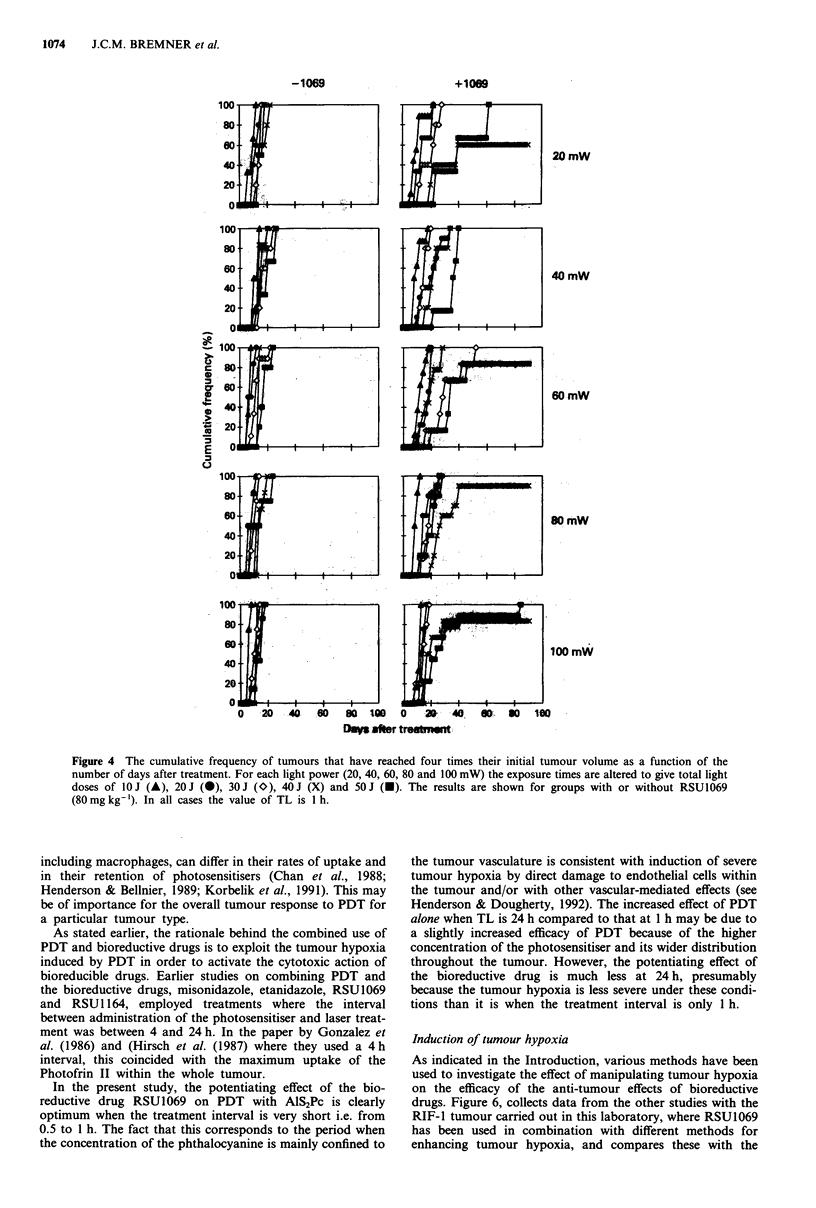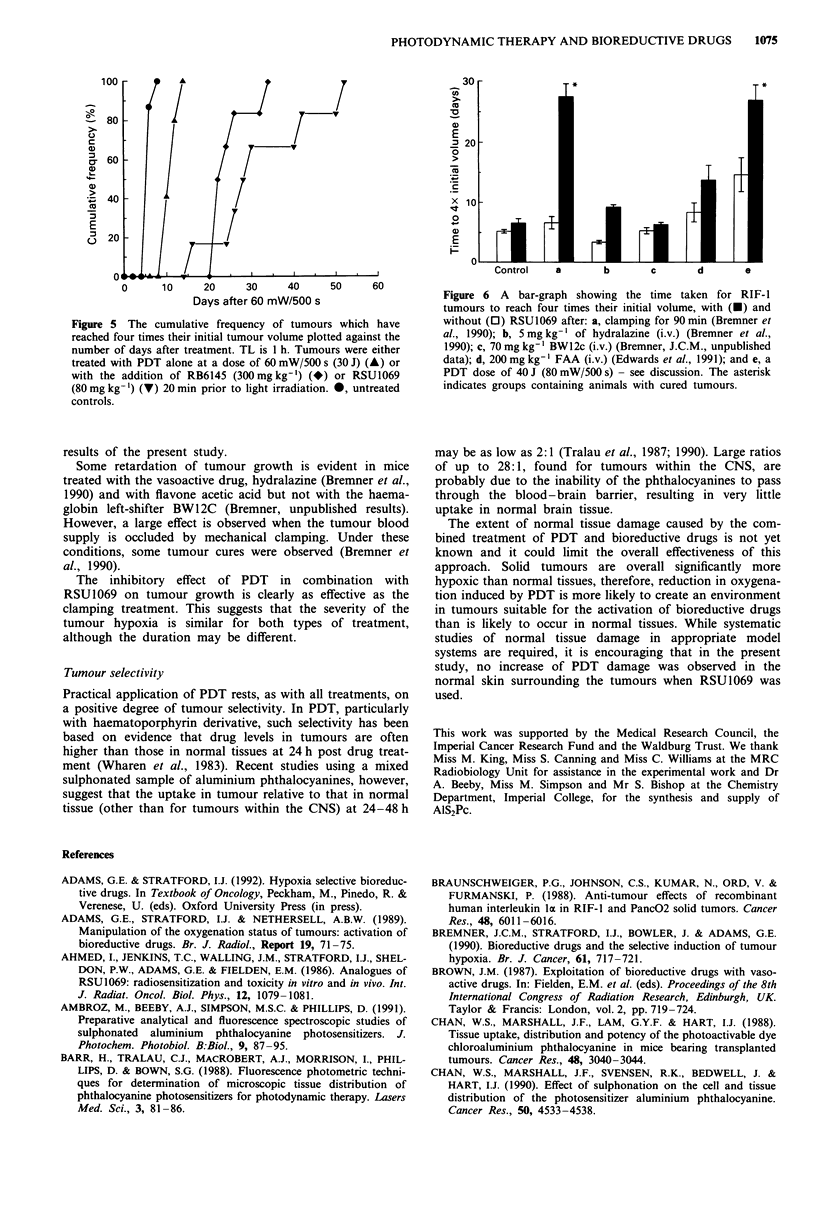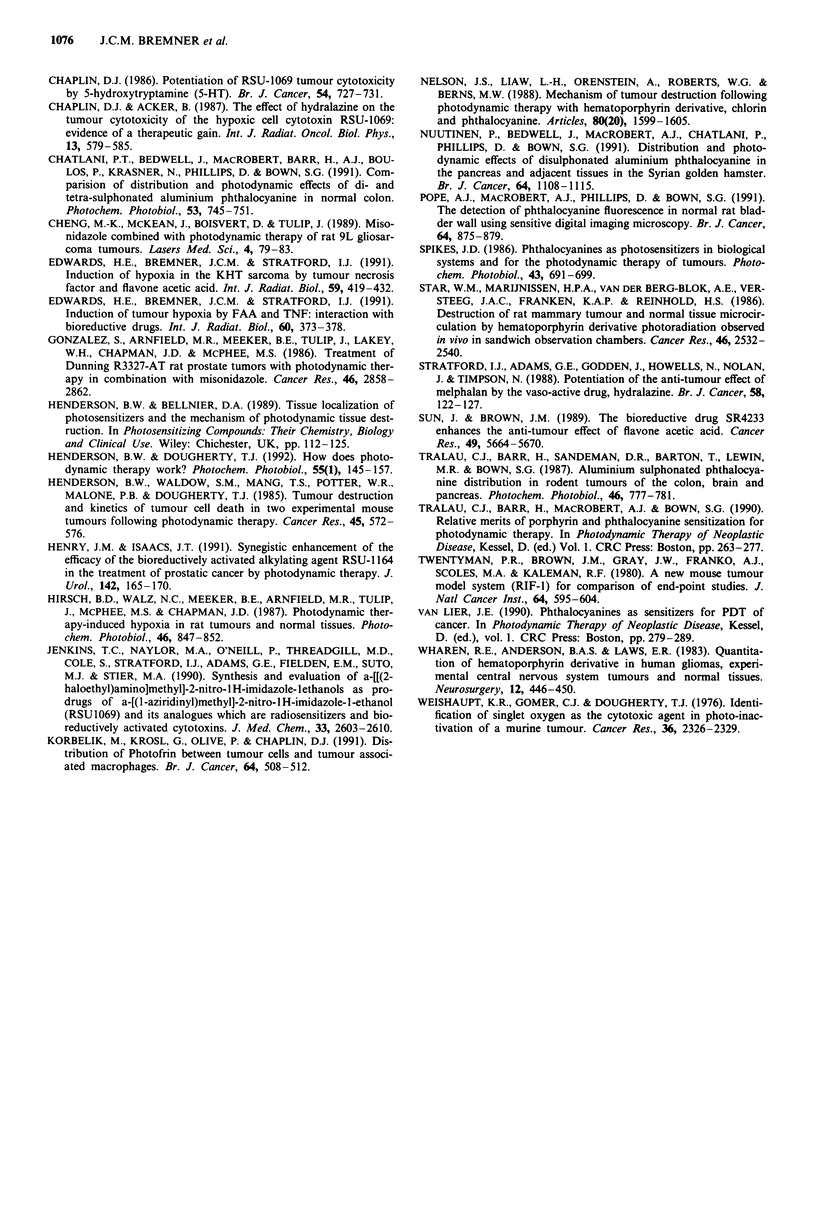# Increasing the effect of photodynamic therapy on the RIF-1 murine sarcoma, using the bioreductive drugs RSU1069 and RB6145.

**DOI:** 10.1038/bjc.1992.412

**Published:** 1992-12

**Authors:** J. C. Bremner, G. E. Adams, J. K. Pearson, J. M. Sansom, I. J. Stratford, J. Bedwell, S. G. Bown, A. J. MacRobert, D. Phillips

**Affiliations:** MRC Radiobiology Unit, Chilton, UK.

## Abstract

**Images:**


*Br. J. Cancer* (1992), **66**, 1070–1076
© Macmillan Press Ltd., 1992

### Increasing the effect of photodynamic therapy on the RIF-1 murine sarcoma, using the bioreductive drugs RSU1069 and RB6145

J.C.M. Bremner[1], G.E. Adams[1], J.K. Pearson[1], J.M. Sansom[1], I.J. Stratford[1], J. Bedwell[2], S.G. Bown[2], A.J. MacRobert[3] & D. Phillips[3]

[1]*MRC Radiobiology Unit, Chilton, Didcot OX11 0RD;* [2]*National Medical Laser Centre, University College and Middlesex School of Medicine, London WC1E 6JJ; and* [3]*Department of Chemistry, Imperial College, London SW7 2AY, UK.*

**Summary** The effect of combining photodynamic therapy (PDT) and bioreductive drugs has been investigated using the RIF-1 experimental murine tumour. Light was delivered interstially to the tumour at 675 nm using a single optical fibre attached to an argon-ion dye laser. The photosensitizer was disulphonated aluminium phthalocyanine ($AlS_2Pc$) and the bioreductive drugs were the dual function nitroimidazole RSU1069 and its pro-drug RB6145. Varying the time between administration of the photosensitizer and light delivery (TL) from 30 min to 24 h had little influence on the extent of the anti-tumour effect of PDT alone, as measured by the regrowth delay endpoint. When the bioreductive drug was included in the treatment, administered 20 min before light irradation, regrowth delay was greatly increased. The effectiveness of the combined treatment was optimum for short values of TL (about 1 h).

Fluorescence microscopy was used to investigate the distribution of the photosensitizer within the tumours. This showed that the compound was mainly confined to the tumour vasculature over the first few hours post-treatment. The high efficacy of the combined treatment of PDT and bioreductive drugs for short values of TL suggest that photodynamic action, during the period when the photosensitizer $AlS_2Pc$ is confined to the vasculature, enhances the severity of tumour hypoxia which is sufficient to induce activation of the bioreductive drugs.

This paper described studies on combining photodynamic therapy (PDT) and bioreductive drugs in the treatment of an experimental murine tumour. Bioreductive drugs are agents that are converted by metabolic reduction to form highly active cytotoxins. These drugs have potential applications in cancer therapy when used with other cytotoxic drugs or with radiotherapy (see Adams & Stratford, 1992).

Since bioreductive drugs are usually activated to a cytotoxic product under hypoxic conditions, their activity can often be enhanced *in vivo* when used together with treatments that enhance the depth and duration of hypoxia in solid tumours. Examples of this approach include the combination of bioreductive drugs with agents that:

1. Reduce blood flow e.g. clamping (Bremner *et al.*, 1990), 5-hydroxytryptamine (Chaplin, 1986) and hydralazine (Brown, 1987; Chaplin & Acker, 1987)
2. Increase the oxygen affinity of haemoglobin e.g. BW12C (Adams *et al.*, 1989).
3. Cause haemorrhagic necrosis, e.g. flavone acetic acid (Sun & Brown, 1989; Edwards *et al.*, 1991), tumour necrosis factor (Edwards *et al.*, 1991) and interleukin-2 (Braunschweiger *et al.*, 1988).

Part of the mechanism of the anti-tumour effect of PDT is believed to be damage to the tumour vasculature (Henderson *et al.*, 1985; Star *et al.*, 1986; Nelson *et al.*, 1988) which causes the reduction in the supply of oxygen and other nutrients to the tumour tissue. A widely proposed mechanistic pathway for cellular destruction with PDT is via the photoactivation of the photosensitiser in the presence of oxygen which results in the generation of highly reactive cytotoxic species including singlet oxygen (Weishaupt *et al.*, 1976; Spikes, 1986; van Lier, 1990).

Chapman and colleagues were the first to use a bioreductive agent (misonidazole) in an attempt to exploit tumour hypoxia induced by PDT. Using a haematoporphyrin derivative (HpD) photosensitizer, they showed that the addition of misonidazole 30 min before, or after, light irradiation caused a significant increase in tumour growth delay and local cure rate in the rat Dunning R3327-AT and R3327-H tumours (Gonzalez *et al.*, 1986; Hirsch *et al.*, 1987). Cheng *et al.* (1989) also used misonidazole in combination with an HpD derivative and laser light in the treatment of rat 9L gliosarcoma tumours. In these experiments, misonidazole was administered 30–45 min before light treatment which was given 24 h after HpD. Misonidazole had no significant effect. The bioreductive drug RSU1069 and its analogue RSU1164, developed in this laboratory (Ahmed *et al.*, 1986) are more potent bioreductive drugs than simple nitroimidazoles due to the incorporation of alkylating functions in the structure. Both RSU1069 (J.D. Chapman and colleagues, unpublished observations) and RSU1164 (Henry & Isaacs, 1991) have been reported to significantly enhance the efficacy of PDT in experimental tumours when using the partly purified form of haemotoporphyrin derivative, Photofrin II, as the photosensitiser.

Recent experimental studies with the second generation photosensitiser, disulphonated aluminium phthalocyanine, $AlS_2Pc$, using fluorescence microscopy, have shown that this photosensitiser is highly concentrated in the vasculature of normal rat colon and bladder 1 h after administration (Chatlani *et al.*, 1991; Pope *et al.*, 1991). This has also been shown for DMH-induced colon tumours in rats (P.T. Chatlani & A.J. MacRobert, unpublished data). This compound is water soluble and has a strong absorption maximum at 675 nm which permits a greater degree of light penetration into tissue than is possible with the 630 nm light used with Photofin II (see Tralau *et al.*, 1990). The majority of clinical studies with PDT employ conditions where the photosensitiser is administered 24–48 h prior to light, however, the highly specific localisation in tissue vasculature during the first hour suggested that light delivered to tumours within this time period after the administration of the photosensitiser might cause more extensive damage to the vasculature. This might induce, therefore, a greater degree of tumour hypoxia than would be achievable if the time period were more prolonged. If so, combined treatment with a bioreductive drug should be more effective when the time between the administration of the photosensitiser and light is short. This hypothesis has been investigated in the RIF-1 tumour using combined PDT with

Correspondence: J.C.M. Bremner, MRC Radiobiology Unit, Chilton, Didcot OX11 0RD, UK.
Received 9 June 1992; and in revised form 21 July 1992

$AlS_2Pc$ and the bioreductive drug RSU1069 and its prodrug RB6145 (Jenkins *et al.*, 1990).

### Materials and methods

### *Tumour models*

The RIF-1 murine sarcoma line was maintained as described previously (Twentyman *et al.*, 1980; Stratford *et al.*, 1988). Approximately $2 \times 10^5$ cells suspended in 0.05 ml PBS were implanted intradermally (i.d.) into the mid-dorsal pelvic region of 8–10 week old C3H/He mice (category IV). The tumour volume at treatment was 100–200 $mm^3$.

Tumours were measured in three orthogonal directions at 2-day intervals. The tumour volume was calculated using the geometric mean of these measurements and assuming spherical geometry. The regrowth endpoint was the time taken for the tumours to grow to four times their volume at the start of treatment.

### *Drugs*

*Bioreductive agents* RSU1069 is an analogue of the nitroimidazole, misonidazole, and contains a weakly basic alkylating aziridine group. A dose of 80 mg $kg^{-1}$ in PBS was used. RB6145 is a haloethylaminohydroxypropyl-2-nitroimidazole which acts as a pro-drug for RSU1069 under physiological conditions. A dose of 300 mg $kg^{-1}$ was administered in acetate buffer (pH 4.5) which minimises its conversion to RSU1069 prior to injection (Jenkins *et al.*, 1990). Both drugs were synthesised by Dr M.A. Naylor from the MRC Radiobiology Unit and administered interperitoneally (i.p.) at 20 ml $kg^{-1}$. The disulphonated aluminium phthalocyanine ($AlS_2Pc$) was synthesised, purified and supplied by the Chemistry Department of Imperial College, London (Ambroz *et al.*, 1991; Nuutinen *et al.*, 1991). This compound was dissolved in isotonic saline and administered intravenously (i.v.). The injection volume was calculated to achieve a final dose in the mouse of 4.37 mg $kg^{-1}$. This represents a dose of 5.7 $\mu$mol $kg^{-1}$ which is the same as that used by Tralau *et al.* (1987) and Chatlani *et al.* (1991).

### *Photodynamic therapy*

*Light source* A Spectra Physics 2016–6 W argon-ion pumped dye-laser was used to generate light at a wavelength of 675 nm. The light was directed down fibre optic cables with a core diameter of 0.2 mm. The ends of these cables were cleaved and inserted interstitially into the centre of the tumours with the fibre positioned parallel to the body of the mouse. As the average diameter of the tumours was approximately 5 mm, only one fibre was used for each tumour. The range of total light doses delivered was 10–50 J. The power density of the light, measured before insertion of the fibre was varied between 20 and 100 mW $cm^{-2}$. Light exposures ranged from 100 to 2,500 s.

*Treatment* The phthalocyanine was administered (i.v.) followed by a dose of light delivered at times (TL) varying from 20 min to 24 h after injection. For the experiments involving RSU1069 and RB6145, these were always given i.p. 20 min before light treatment. This time sequence allowed uptake of bioreductive drugs in the RIF-1 tumour to reach maximum levels before light treatment. The mice were anaesthetised 0.1 ml injections (i.p.) of a 1:1:2 mixture of Hypnorm:Hypnovel:water 10 min before the administration of the light. One ml of Hypnovel contains 10 mg midazolam base as the hydrochloride. Hypnorm contains fentanyl citrate at 0.315 mg $ml^{-1}$ and fluanison at 10 mg $ml^{-1}$. After treatment all animals were carefully wrapped in aluminium foil and tissue paper to restrict the amount of body-heat loss which may be induced by the anaesthetic and/or by the bioreductive drug.

### *Histology and fluorescence microscopy*

In order to determine the uptake and distribution of the photosensitiser in the RIF-1 sarcoma, tumours were excised post mortem at various times after the administration of $AlS_2Pc$. They were snap frozen in liquid nitrogen, and two serial cryostat sections were taken (10 $\mu$m slices) from each tumour, one section for staining with haematoxylin and eosin and the other for fluorescence microscopy analysis using a cooled charge-coupled device (CCD) (Barr *et al.*, 1988; Pope *et al.*, 1991). A slightly modified technique was used as described by Chatlani *et al.* (1991).

### Results

### *Photosensitiser distribution in RIF-1 tumours*

As discussed earlier, the design of this study was based on the hypothesis that PDT delivered whilst the sensitiser is confined mainly to the vasculature, would induce a more severe degree of hypoxia and therefore should aid the effectiveness of the bioreductive agent. Some experiments were carried out using fluorescence microscopy (CCD) in the RIF tumours of the size range of those used for the PDT experiments. A series of fluorescent micrographs was prepared for tumours from mice given $AlS_2Pc$ at times varying from 5 min to 24 h after the administration of the photosensitiser. (Two mice were used for each time point.) Examples of fluoromicrographs for times of 30 min (Figure 1a), 6 h (Figure 1c) and 24 h (Figure 1e) are compared with serial sections of the same tumour samples stained with haematoxylin and eosin (Figure 1b,d,f). At 30 min, the fluorescence is very bright and appears to originate mainly within blood vessels, with a small amount of fluorescence evident in the perivascular regions. At 6 h the fluorescence is still mainly within the blood vessels although there is more fluorescence diffusely spread throughout the tumour. At 24 h the total level of fluorescence from the frozen sections is about 2–3 times greater than at 30 min. However, the blood vessels are no longer prominent indicating that there is little or no photosensitiser remaining within the vasculature. The fluorescence appears to be associated with the surrounding tissue, particularly within the necrotic regions.

### *Regrowth delay studies*

No effect on the tumour growth is observed for any of the control groups as shown in Table I. Figure 2 shows regrowth delay data for tumours treated with PDT at a light dose of 50 J (100 mW/500 s) given from 1 to 24 h after administration of $AlS_2Pc$. The overall effect on tumour growth shows little dependence on the value of TL. However when TL is 1 h there is an initial sharp drop in tumour volume which is not evident in the other growth curves. This size decrease appears to have little effect on subsequent growth rate. For TL of 1 h, severe scabbing occurs covering the whole tumour. This is not observed for values of TL greater than 1 h.

There is no effect of RSU1069 on tumour response when given alone or in combination with either $AlS_2Pc$ alone *or* with light alone (100 mW/500 s) in anaesthetised mice (Table I). Figure 3 shows the effect of RSU1069, on tumour response to PDT. When TL is 24 h, tumour growth is further retarded relative to that observed for PDT alone when RSU1069 is included in the treartment, although the effect is relatively small. In contrast, when TL is reduced to 1 h, this increased tumour delay induced by RSU1069 is substantially greater.

Table II shows, for a range of values of TL, the effect on tumour growth, expressed as the time taken for the tumours to reach 4 times their initial treatment volume. The light dose was maintained constant at 100 mW/500 s. RSU1069 potentiates the PDT in all cases but is most effective when TL is 30 min or 1 h. Indeed for the 1 h point, two out of the group of eight tumours showed very long regrowth delays (up to 83

**Figure 1** Examples of fluorescence micrographs (10 μm) of RIF-1 tumours imaged using a CCD device are shown at 30 min **a**, 6 h **c**, and 24 h **e**, after the administration of $AlS_2Pc$. The colour scale is shown at the top of the micrograph with the maximum fluorescence being represented as white and the minimum as black. Serial sections of the same tumours stained with haematoxylin and eosin are also showed (**b**, **d**, **f**). The boxes delineate identifiable blood vessels.

days). This accounts for the large range in delay values for this group as is shown in Table II.

It is possible to vary the total light dose (TD) and dose rate by changing the parameters of the light source power (P) and total treatment time (T) since TD = P × T. For power values of 20, 40, 60, 80 or 100 mW, exposure times varying from 100 to 2,500 s were used to obtain a range in total doses of 10–50 J. Results showing the effect of RSU1069, when TL is 1 h, are plotted as cumulative frequency curves in Figure 4. This shows the percentage of tumours that have grown to four times their initial treatment volume plotted against the number of days after treatment. This type of plot allows all the animals in a group to be used in the analysis, even those that are 'cured' i.e. that have no evidence of local tumour recurrence up to 160 days after treatment. Each plot contains data from a minimum of six animals. In the groups receiving PDT alone, for each value of power, an increase in the total dose from 10 J to 50 J causes a small but observable increase in effect. However, when RSU1069 is administered 20 min before light exposure, the growth of the tumour is greatly retarded for total light doses in excess of 30 J. Some long-term cures (up to 33.3%) were obtained in groups treated with doses of 30–50 J. There was no trend showing any influence of dose rate over the power range of 20–100 mW either with, or without, RSU1069.

For comparison, the potentiating effect of RB6145 was investigated for a total light dose of 30 J (60 mW/500 s). The cumulative frequency data in Figure 5 show that this agent, when administered at its maximum tolerated dose (MTD) of 300 mg $kg^{-1}$ (i.p.), also substantially enhances PDT.

### Discussion

The following main conclusions can be drawn from the results of this study:

**Figure 2** Tumour growth curves showing the effect of varying TL in groups of animals receiving a PDT dose of 100 mW/500 s. Mean tumour size (expressed as GMD) ± s.e.m. is plotted against the number of days after treatment.

**Figure 3** Tumour growth curves showing the increased effect induced by RSU1069 (80 mg $kg^{-1}$) when administered 20 min prior to a light irradiation dose of 100 mW/500 s. The results are shown for groups with a TL of 1 h (□ ■) and 24 h (△ ▲). Closed and open symbols represent data from groups with PDT alone and PDT + RSU1069 respectively.

1. $AlS_2Pc$ is an effective photosensitiser for the RIF-1 tumour when used at a dose of 4.37 mg $kg^{-1}$ administered from 20 min to 24 h before laser treatment.
2. With PDT alone, for a dose of 50 J, there is little effect of varying TL from 1 h to 24 h on the efficacy of treatment although the effect is significantly greater for a shorter delay time of 30 min.
3. RSU1069 greatly potentiates the effect of PDT for values of TL between 30 min and 6 h. RB6145, a prodrug for RSU1069 also potentiates PDT.
4. For the optimum value of TL of 1 h, some tumour cures were obtained in groups of mice treated with PDT and RSU1069 at light doses of 30 J and above.
5. There is no significant trend with dose rate over the power range 20–100 mW.

*Distribution kinetics of the photosensitiser within the tumour*

There is a 2–3 fold increase in total fluorescence from the tumour sections taken between 30 min and 24 h after injection, which indicates a gradual build up of the photosensitiser within the tumour tissue. The histological preparations in Figure 1 show that the distribution of the photosensitiser between the vascular and perivascular regions varied considerably over a 24 h period. Between 30 min and 6 h, the drug appears to be mainly confined within the vasculature of the tumour. This is consistent with previous findings showing that $AlS_2Pc$ is at maximum levels within blood vessels of the colon (Chatlani *et al.*, 1991) and the DMH-induced colonic rat tumours (P.T. Chatlani & A.J. MacRobert, unpublished data) at 1 h after administration. The fluorescent micrographs in Figure 1 show that in the RIF-1 tumour there is little or no photosensitiser detectable within the blood vessels at 24 h post-administration. This data agrees with that of Chan *et al.* (1990) who showed a 10-fold reduction of $AlS_2Pc$ in the plasma from 3 to 24 h after administration. Although it is not possible, from these micrographs, to determine precisely in which cells in the RIF-1 tumour the $AlS_2Pc$ is located at 24 h, there is evidence that cells of different types,

**Table I** Time in days for control tumours to reach four times their initial volume in anaesthetised mice. The numbers of mice and the range in values are indicated for each group

| | *No RSU1069* | | | *+RSU1069* | | |
|---|---|---|---|---|---|---|
| *TL* | *Growth time ± s.e.* | *Nos.* | *Range* | *Growth time ± s.e.* | *Nos.* | *Range* |
| Untreated control | 5.4±0.2 | 10 | 4.8–6.1 | 6.2±0.6 | 6 | 5.1–8.5 |
| Anaesthetised control | 5.2±0.3 | 10 | 4.3–7.3 | 6.6±0.7 | 7 | 4.6–9.3 |
| $AlS_2Pc$ alone | 6.1±0.8 | 8 | 3.2–8.5 | 5.6±0.6 | 6 | 4.8–6.9 |
| Light alone 100 mW/500 s | 6.2±0.2 | 6 | 5.7–7.1 | 6.1±0.5 | 6 | 5.6–7.0 |

**Table II** Time in days for tumours to reach four times their initial volume as a function of the time between administration of the photosensitiser and the light irradiation (TL). The numbers of mice and the range in values are given for each group. Light doses in all cases was 100 mW for 500 s

| | *No RSU1069* | | | *+RSU1069* | | |
|---|---|---|---|---|---|---|
| *TL* | *Growth time ± s.e.* | *Nos.* | *Range* | *Growth time ± s.e.* | *Nos.* | *Range* |
| Anaethetised control | 5.2±0.3 | 10 | 4.3–7.3 | 6.6±0.7 | 7 | 4.6–9.3 |
| 20 min | 19.1±1.0 | 6 | 16.1–22.4 | 20.0±1.2 | 8 | 16.5–26.5 |
| 30 min | 23.1±1.0 | 11 | 15.2–33.6 | 35.4±1.2 | 10 | 25.9–44.9 |
| 1 h | 15.0±1.2 | 7 | 9.7–18.3 | 34.3±12.7 | 8 | 14.5–83.2 |
| 2 h | 12.6±1.0 | 6 | 8.6–15.7 | 24.2±2.3 | 6 | 15.1–30.0 |
| 4 h | 11.2±0.4 | 6 | 9.3±12.6 | 21.3±1.6 | 12 | 12.4–27.9 |
| 6 h | 11.3±0.4 | 6 | 10.3–13.3 | 19.9±1.4 | 6 | 13.1–22.8 |
| 24 h | 17.9±1.7 | 10 | 10.7–29.9 | 21.1±1.0 | 15 | 15.1–29.9 |

**Figure 4** The cumulative frequency of tumours that have reached four times their initial tumour volume as a function of the number of days after treatment. For each light power (20, 40, 60, 80 and 100 mW) the exposure times are altered to give total light doses of 10 J (▲), 20 J (●), 30 J (◇), 40 J (X) and 50 J (■). The results are shown for groups with or without RSU1069 (80 mg $kg^{-1}$). In all cases the value of TL is 1 h.

including macrophages, can differ in their rates of uptake and in their retention of photosensitisers (Chan *et al.*, 1988; Henderson & Bellnier, 1989; Korbelik *et al.*, 1991). This may be of importance for the overall tumour response to PDT for a particular tumour type.

As stated earlier, the rationale behind the combined use of PDT and bioreductive drugs is to exploit the tumour hypoxia induced by PDT in order to activate the cytotoxic action of bioreducible drugs. Earlier studies on combining PDT and the bioreductive drugs, misonidazole, etanidazole, RSU1069 and RSU1164, employed treatments where the interval between administration of the photosensitiser and laser treatment was between 4 and 24 h. In the paper by Gonzalez *et al.* (1986) and (Hirsch *et al.* (1987) where they used a 4 h interval, this coincided with the maximum uptake of the Photofrin II within the whole tumour.

In the present study, the potentiating effect of the bioreductive drug RSU1069 on PDT with $AlS_2Pc$ is clearly optimum when the treatment interval is very short i.e. from 0.5 to 1 h. The fact that this corresponds to the period when the concentration of the phthalocyanine is mainly confined to the tumour vasculature is consistent with induction of severe tumour hypoxia by direct damage to endothelial cells within the tumour and/or with other vascular-mediated effects (see Henderson & Dougherty, 1992). The increased effect of PDT *alone* when TL is 24 h compared to that at 1 h may be due to a slightly increased efficacy of PDT because of the higher concentration of the photosensitiser and its wider distribution throughout the tumour. However, the potentiating effect of the bioreductive drug is much less at 24 h, presumably because the tumour hypoxia is less severe under these conditions than it is when the treatment interval is only 1 h.

### *Induction of tumour hypoxia*

As indicated in the Introduction, various methods have been used to investigate the effect of manipulating tumour hypoxia on the efficacy of the anti-tumour effects of bioreductive drugs. Figure 6, collects data from the other studies with the RIF-1 tumour carried out in this laboratory, where RSU1069 has been used in combination with different methods for enhancing tumour hypoxia, and compares these with the

**Figure 5** The cumulative frequency of tumours which have reached four times their initial tumour volume plotted against the number of days after treatment. TL is 1 h. Tumours were either treated with PDT alone at a dose of 60 mW/500 s (30 J) (▲) or with the addition of RB6145 (300 mg $kg^{-1}$) (◆) or RSU1069 (80 mg $kg^{-1}$) (▼) 20 min prior to light irradiation. ●, untreated controls.

**Figure 6** A bar-graph showing the time taken for RIF-1 tumours to reach four times their initial volume, with (■) and without (□) RSU1069 after: **a**, clamping for 90 min (Bremner *et al.*, 1990); **b**, 5 mg $kg^{-1}$ of hydralazine (i.v.) (Bremner *et al.*, 1990); **c**, 70 mg $kg^{-1}$ BW12c (i.v.) (Bremner, J.C.M., unpublished data); **d**, 200 mg $kg^{-1}$ FAA (i.v.) (Edwards *et al.*, 1991); and **e**, a PDT dose of 40 J (80 mW/500 s) – see discussion. The asterisk indicates groups containing animals with cured tumours.

results of the present study.

Some retardation of tumour growth is evident in mice treated with the vasoactive drug, hydralazine (Bremner *et al.*, 1990) and with flavone acetic acid but not with the haemaglobin left-shifter BW12C (Bremner, unpublished results). However, a large effect is observed when the tumour blood supply is occluded by mechanical clamping. Under these conditions, some tumour cures were observed (Bremner *et al.*, 1990).

The inhibitory effect of PDT in combination with RSU1069 on tumour growth is clearly as effective as the clamping treatment. This suggests that the severity of the tumour hypoxia is similar for both types of treatment, although the duration may be different.

### *Tumour selectivity*

Practical application of PDT rests, as with all treatments, on a positive degree of tumour selectivity. In PDT, particularly with haematoporphyrin derivative, such selectivity has been based on evidence that drug levels in tumours are often higher than those in normal tissues at 24 h post drug treatment (Wharen *et al.*, 1983). Recent studies using a mixed sulphonated sample of aluminium phthalocyanines, however, suggest that the uptake in tumour relative to that in normal tissue (other than for tumours within the CNS) at 24–48 h may be as low as 2:1 (Tralau *et al.*, 1987; 1990). Large ratios of up to 28:1, found for tumours within the CNS, are probably due to the inability of the phthalocyanines to pass through the blood–brain barrier, resulting in very little uptake in normal brain tissue.

The extent of normal tissue damage caused by the combined treatment of PDT and bioreductive drugs is not yet known and it could limit the overall effectiveness of this approach. Solid tumours are overall significantly more hypoxic than normal tissues, therefore, reduction in oxygenation induced by PDT is more likely to create an environment in tumours suitable for the activation of bioreductive drugs than is likely to occur in normal tissues. While systematic studies of normal tissue damage in appropriate model systems are required, it is encouraging that in the present study, no increase of PDT damage was observed in the normal skin surrounding the tumours when RSU1069 was used.

This work was supported by the Medical Research Council, the Imperial Cancer Research Fund and the Waldburg Trust. We thank Miss M. King, Miss S. Canning and Miss C. Williams at the MRC Radiobiology Unit for assistance in the experimental work and Dr A. Beeby, Miss M. Simpson and Mr S. Bishop at the Chemistry Department, Imperial College, for the synthesis and supply of $AlS_2Pc$.

### References

ADAMS, G.E. & STRATFORD, I.J. (1992). Hypoxia selective bioreductive drugs. In *Textbook of Oncology*, Peckham, M., Pinedo, R. & Verenese, U. (eds). Oxford University Press (in press).

ADAMS, G.E., STRATFORD, I.J. & NETHERSELL, A.B.W. (1989). Manipulation of the oxygenation status of tumours: activation of bioreductive drugs. *Br. J. Radiol.*, **Report 19,** 71–75.

AHMED, I., JENKINS, T.C., WALLING, J.M., STRATFORD, I.J., SHELDON, P.W., ADAMS, G.E. & FIELDEN, E.M. (1986). Analogues of RSU1069: radiosensitization and toxicity *in vitro* and *in vivo*. *Int. J. Radiat. Oncol. Biol. Phys.*, **12,** 1079–1081.

AMBROZ, M., BEEBY, A.J., SIMPSON, M.S.C. & PHILLIPS, D. (1991). Preparative analytical and fluorescence spectroscopic studies of sulphonated aluminium phthalocyanine photosensitizers. *J. Photochem. Photobiol. B:Biol.*, **9,** 87–95.

BARR, H., TRALAU, C.J., MACROBERT, A.J., MORRISON, I., PHILLIPS, D. & BOWN, S.G. (1988). Fluorescence photometric techniques for determination of microscopic tissue distribution of phthalocyanine photosensitizers for photodynamic therapy. *Lasers Med. Sci.*, **3,** 81–86.

BRAUNSCHWEIGER, P.G., JOHNSON, C.S., KUMAR, N., ORD, V. & FURMANSKI, P. (1988). Anti-tumour effects of recombinant human interleukin 1α in RIF-1 and PancO2 solid tumors. *Cancer Res.*, **48,** 6011–6016.

BREMNER, J.C.M., STRATFORD, I.J., BOWLER, J. & ADAMS, G.E. (1990). Bioreductive drugs and the selective induction of tumour hypoxia. *Br. J. Cancer*, **61,** 717–721.

BROWN, J.M. (1987). Exploitation of bioreductive drugs with vasoactive drugs. In: Fielden, E.M. *et al.* (eds). *Proceedings of the 8th International Congress of Radiation Research, Edinburgh, UK.* Taylor & Francis: London, vol. 2, pp. 719–724.

CHAN, W.S., MARSHALL, J.F., LAM, G.Y.F. & HART, I.J. (1988). Tissue uptake, distribution and potency of the photoactivable dye chloroaluminium phthalocyanine in mice bearing transplanted tumours. *Cancer Res.*, **48,** 3040–3044.

CHAN, W.S., MARSHALL, J.F., SVENSEN, R.K., BEDWELL, J. & HART, I.J. (1990). Effect of sulphonation on the cell and tissue distribution of the photosensitizer aluminium phthalocyanine. *Cancer Res.*, **50,** 4533–4538.

CHAPLIN, D.J. (1986). Potentiation of RSU-1069 tumour cytotoxicity by 5-hydroxytryptamine (5-HT). *Br. J. Cancer*, **54**, 727-731.

CHAPLIN, D.J. & ACKER, B. (1987). The effect of hydralazine on the tumour cytotoxicity of the hypoxic cell cytotoxin RSU-1069: evidence of a therapeutic gain. *Int. J. Radiat. Oncol. Biol. Phys.*, **13**, 579-585.

CHATLANI, P.T., BEDWELL, J., MACROBERT, BARR, H., A.J., BOULOS, P., KRASNER, N., PHILLIPS, D. & BOWN, S.G. (1991). Comparision of distribution and photodynamic effects of di- and tetra-sulphonated aluminium phthalocyanine in normal colon. *Photochem. Photobiol.*, **53**, 745-751.

CHENG, M.-K., MCKEAN, J., BOISVERT, D. & TULIP, J. (1989). Misonidazole combined with photodynamic therapy of rat 9L gliosarcoma tumours. *Lasers Med. Sci.*, **4**, 79-83.

EDWARDS, H.E., BREMNER, J.C.M. & STRATFORD, I.J. (1991). Induction of hypoxia in the KHT sarcoma by tumour necrosis factor and flavone acetic acid. *Int. J. Radiat. Biol.*, **59**, 419-432.

EDWARDS, H.E., BREMNER, J.C.M. & STRATFORD, I.J. (1991). Induction of tumour hypoxia by FAA and TNF: interaction with bioreductive drugs. *Int. J. Radiat. Biol.*, **60**, 373-378.

GONZALEZ, S., ARNFIELD, M.R., MEEKER, B.E., TULIP, J., LAKEY, W.H., CHAPMAN, J.D. & MCPHEE, M.S. (1986). Treatment of Dunning R3327-AT rat prostate tumors with photodynamic therapy in combination with misonidazole. *Cancer Res.*, **46**, 2858-2862.

HENDERSON, B.W. & BELLNIER, D.A. (1989). Tissue localization of photosensitizers and the mechanism of photodynamic tissue destruction. In *Photosensitizing Compounds: Their Chemistry, Biology and Clinical Use*. Wiley: Chichester, UK, pp. 112-125.

HENDERSON, B.W. & DOUGHERTY, T.J. (1992). How does photodynamic therapy work? *Photochem. Photobiol.*, **55(1)**, 145-157.

HENDERSON, B.W., WALDOW, S.M., MANG, T.S., POTTER, W.R., MALONE, P.B. & DOUGHERTY, T.J. (1985). Tumour destruction and kinetics of tumour cell death in two experimental mouse tumours following photodynamic therapy. *Cancer Res.*, **45**, 572-576.

HENRY, J.M. & ISAACS, J.T. (1991). Synegistic enhancement of the efficacy of the bioreductively activated alkylating agent RSU-1164 in the treatment of prostatic cancer by photodynamic therapy. *J. Urol.*, **142**, 165-170.

HIRSCH, B.D., WALZ, N.C., MEEKER, B.E., ARNFIELD, M.R., TULIP, J., MCPHEE, M.S. & CHAPMAN, J.D. (1987). Photodynamic therapy-induced hypoxia in rat tumours and normal tissues. *Photochem. Photobiol.*, **46**, 847-852.

JENKINS, T.C., NAYLOR, M.A., O'NEILL, P., THREADGILL, M.D., COLE, S., STRATFORD, I.J., ADAMS, G.E., FIELDEN, E.M., SUTO, M.J. & STIER, M.A. (1990). Synthesis and evaluation of a-[[(2-haloethyl)amino]methyl]-2-nitro-1H-imidazole-1ethanols as prodrugs of a-[(1-aziridinyl)methyl]-2-nitro-1H-imidazole-1-ethanol (RSU1069) and its analogues which are radiosensitizers and bioreductively activated cytotoxins. *J. Med. Chem.*, **33**, 2603-2610.

KORBELIK, M., KROSL, G., OLIVE, P. & CHAPLIN, D.J. (1991). Distribution of Photofrin between tumour cells and tumour associated macrophages. *Br. J. Cancer*, **64**, 508-512.

NELSON, J.S., LIAW, L.-H., ORENSTEIN, A., ROBERTS, W.G. & BERNS, M.W. (1988). Mechanism of tumour destruction following photodynamic therapy with hematoporphyrin derivative, chlorin and phthalocyanine. *Articles*, **80(20)**, 1599-1605.

NUUTINEN, P., BEDWELL, J., MACROBERT, A.J., CHATLANI, P., PHILLIPS, D. & BOWN, S.G. (1991). Distribution and photodynamic effects of disulphonated aluminium phthalocyanine in the pancreas and adjacent tissues in the Syrian golden hamster. *Br. J. Cancer*, **64**, 1108-1115.

POPE, A.J., MACROBERT, A.J., PHILLIPS, D. & BOWN, S.G. (1991). The detection of phthalocyanine fluorescence in normal rat bladder wall using sensitive digital imaging microscopy. *Br. J. Cancer*, **64**, 875-879.

SPIKES, J.D. (1986). Phthalocyanines as photosensitizers in biological systems and for the photodynamic therapy of tumours. *Photochem. Photobiol.*, **43**, 691-699.

STAR, W.M., MARIJNISSEN, H.P.A., VAN DER BERG-BLOK, A.E., VERSTEEG, J.A.C., FRANKEN, K.A.P. & REINHOLD, H.S. (1986). Destruction of rat mammary tumour and normal tissue microcirculation by hematoporphyrin derivative photoradiation observed *in vivo* in sandwich observation chambers. *Cancer Res.*, **46**, 2532-2540.

STRATFORD, I.J., ADAMS, G.E., GODDEN, J., HOWELLS, N., NOLAN, J. & TIMPSON, N. (1988). Potentiation of the anti-tumour effect of melphalan by the vaso-active drug, hydralazine. *Br. J. Cancer*, **58**, 122-127.

SUN, J. & BROWN, J.M. (1989). The bioreductive drug SR4233 enhances the anti-tumour effect of flavone acetic acid. *Cancer Res.*, **49**, 5664-5670.

TRALAU, C.J., BARR, H., SANDEMAN, D.R., BARTON, T., LEWIN, M.R. & BOWN, S.G. (1987). Aluminium sulphonated phthalocyanine distribution in rodent tumours of the colon, brain and pancreas. *Photochem. Photobiol.*, **46**, 777-781.

TRALAU, C.J., BARR, H., MACROBERT, A.J. & BOWN, S.G. (1990). Relative merits of porphyrin and phthalocyanine sensitization for photodynamic therapy. In *Photodynamic Therapy of Neoplastic Disease*, Kessel, D. (ed.) Vol. 1. CRC Press: Boston, pp. 263-277.

TWENTYMAN, P.R., BROWN, J.M., GRAY, J.W., FRANKO, A.J., SCOLES, M.A. & KALEMAN, R.F. (1980). A new mouse tumour model system (RIF-1) for comparison of end-point studies. *J. Natl Cancer Inst.*, **64**, 595-604.

VAN LIER, J.E. (1990). Phthalocyanines as sensitizers for PDT of cancer. In *Photodynamic Therapy of Neoplastic Disease*, Kessel, D. (ed.), vol. 1. CRC Press: Boston, pp. 279-289.

WHAREN, R.E., ANDERSON, B.A.S. & LAWS, E.R. (1983). Quantitation of hematoporphyrin derivative in human gliomas, experimental central nervous system tumours and normal tissues. *Neurosurgery*, **12**, 446-450.

WEISHAUPT, K.R., GOMER, C.J. & DOUGHERTY, T.J. (1976). Identification of singlet oxygen as the cytotoxic agent in photo-inactivation of a murine tumour. *Cancer Res.*, **36**, 2326-2329.